# Supplementary material for: From time-inconsistency to time-consistency for optimal stopping problems
Source: PLoS One. 2024 Nov 12;19(11):e0310774. doi: 10.1371/journal.pone.0310774 (PMC11556735; doi:10.1371/journal.pone.0310774)
Supplement: S1 Code — This file explains how to generate strategies and figures in the examples in Section 4. (PDF) [file pone.0310774.s001.pdf]

Description of Code

We employ the nonlinear optimization solver *"fmincon"* from MATLAB Optimization Toolbox and global minimization solver *"surrogateopt"* from MATLAB Global Optimization Toolbox to solve the optimal stopping problem in a binomial tree model.

1 Initial Setting and Parameters

We adopt the following settings for *"fmincon"*:

```
rng default      For reproducibility
ms = MultiStart('UseParallel',true);
gs = GlobalSearch(ms);
```

Also, we give the notations of the parameters used in our code:

- *T*: period of the binomial tree
- *alpha_pos*/*alpha_neg*: parameters in the utility function
- *lambda*: degree of loss aversion in the utility function
- *delta_pos*/*delta_neg*: parameters in the probability weighting function

Consider a 5-period binomial tree with two different sets of parameters in the utility function and probability weighting function:

```
% utility function parameter
alpha_pos = 0.9;    % alpha_pos in (0,1)
alpha_neg = 0.9;    % alpha_neg in (0,1)
lambda = 1.5;           % lambda > 1
% weighting function parameter
delta_pos = 0.5;    % delta_pos in (0,1)
delta_neg = 0.5;    % delta_neg in (0,1)
```

```
% utility function parameter
alpha_pos = 0.5;    % alpha_pos in (0,1)
alpha_neg = 0.5;    % alpha_neg in (0,1)
lambda = 1.5;           % lambda > 1
% weighting function parameter
delta_pos = 0.9;    % delta_pos in (0,1)
delta_neg = 0.9;    % delta_neg in (0,1)
```

2 Definition of Functions

Given a *T*-period binomial tree and stopping strategy *"p"*, which contains the stopping probability at each node, function *"tree_built"* outputs all the possible final wealth in *state_space.value* and the corresponding probability of stopping at a certain wealth in *state_space.prob* starting from time *t0* and state *x0*.

```
function [binomial_tree, state_space] = tree_built(t0, x0, T, p)
% Input        (t0, x0)             starting pair
% Input        T                    period of binomial tree
% Input        p                    stopping probability at each node
% Output       binomial_tree        time t in [t0 T]
%                                   state x in [x0-(T-t0) x0+(T-t0)]
%                                   stopping/continue probability at node (t, x)
% Output       state_space          values in [x0-(T-t0) x0+(T-t0)]
%                                   probability of stopping at each state

binomial_tree = struct();

% probability of stopping at each state
state_space = struct();
state_space.value = x0-(T-t0):x0+(T-t0);
state_space.prob = zeros(1,length(state_space.value));

for i = 1:T-t0+1
    % time t
    binomial_tree(i).time = i+t0-1;
    for n = 1:i
        % states at time t0+i-1 (from highest to lowest)
        state = x0+(i-1)-2*(n-1);
        binomial_tree(i).state(n) = state;
        % stopping probability at node (t0+i-1,state)
        if binomial_tree(i).time == T
            binomial_tree(i).stopping_strategy(n) = 1;
        else
            binomial_tree(i).stopping_strategy(n) = p(i*(i-1)/2+n);
        end
        % probability of stopping/continue at node (t0+i-1, state)
        if binomial_tree(i).time == t0
            binomial_tree(i).stopping_prob(n) = binomial_tree(i).stopping_strategy(n);
            binomial_tree(i).continue_prob(n) = 1 - binomial_tree(i).stopping_prob(n);
```

```
            else
                if    n > 1 && n < i
                    % there are upward (i-1,n) and downward (i-1,n-1) paths to node (i, n)
                    binomial_tree(i).stopping_prob(n) = 0.5 *
binomial_tree(i).stopping_strategy(n)...
                        * (binomial_tree(i-1).continue_prob(n-1) + binomial_tree(i-
1).continue_prob(n));
                    binomial_tree(i).continue_prob(n) = 0.5 * (1-
binomial_tree(i).stopping_strategy(n))...
                        * (binomial_tree(i-1).continue_prob(n-1) + binomial_tree(i-
1).continue_prob(n));
                elseif n == 1
                    % there only have upward path from node (i-1,1) to (i, 1)
                    binomial_tree(i).stopping_prob(n) = 0.5 *
binomial_tree(i).stopping_strategy(n)...
                        * binomial_tree(i-1).continue_prob(n);
                    binomial_tree(i).continue_prob(n) = 0.5 * (1-
binomial_tree(i).stopping_strategy(n))...
                        * binomial_tree(i-1).continue_prob(n);
                elseif n == i
                    % there only have downward path from (i-1,n-1) to node (i,n)
                    binomial_tree(i).stopping_prob(n) = 0.5 *
binomial_tree(i).stopping_strategy(n)...
                        * binomial_tree(i-1).continue_prob(n-1);
                    binomial_tree(i).continue_prob(n) = 0.5 * (1-
binomial_tree(i).stopping_strategy(n))...
                        * binomial_tree(i-1).continue_prob(n-1);
                end
            end

            for s = 1:length(state_space.value)
                if state_space.value(s) == binomial_tree(i).state(n)
                    state_space.prob(s) = state_space.prob(s) +
binomial_tree(i).stopping_prob(n);
                    break
                end
            end
        end
end
end
```

We then define the utility function *“utility”* and probability weighting function *“weighting”* under the cumulative prospect theory.

```
function [u] = utility(x, lambda, alpha_pos, alpha_neg)
% utility function
% Input       x                       wealth
% Output      u                       corresponding utility
if x >= 0
      u = x^alpha_pos;
else
      u = -lambda*(-x)^alpha_neg;
end
end
```

```
function [w] = weighting(sign, delta_pos, delta_neg, pr)
% probability weighting function
% Input       sign               the sign of wealth: 'pos' or 'neg'
% Input       pr                 probability
% Output      w                  weighting probability
if sign == 'pos'
      w = pr^delta_pos/((pr^delta_pos+(1-pr)^delta_pos)^(1/delta_pos));
elseif sign == 'neg'
      w = pr^delta_neg/((pr^delta_neg+(1-pr)^delta_neg)^(1/delta_neg));
end
end
```

Based on the utility function *“utility”* and probability weighting function *“weighting”*, function *“objective”* calculates the CPT preference value for a given stopping strategy *“p”* at time *t0* and state *x0* in a *T*-period binomial tree, where the probability of stopping at a certain wealth is directly obtained by using the function *“tree_built”*.

```
function [objective] = objective(lambda, alpha_pos, alpha_neg, delta_pos, delta_neg, t0, x0, T,
p)
% CPT preference value
[~, state_space] = tree_built(t0, x0, T, p);

objective = 0;    % initial setting
x = state_space.value;
pr = state_space.prob;
```

```
for i = 1:length(x)
    if x(i) > 0
        if i == length(x)
            objective = objective + utility(x(i), lambda, alpha_pos, alpha_neg)...
                * weighting('pos',delta_pos,delta_neg,pr(i));
        else
            objective = objective + utility(x(i), lambda, alpha_pos, alpha_neg)...
                * (weighting('pos',delta_pos,delta_neg,sum(pr(i:length(x))))...
                - weighting('pos',delta_pos,delta_neg,sum(pr(i+1:length(x)))));
        end
    elseif x(i) < 0
        if i == 1
            objective = objective + utility(x(i), lambda, alpha_pos, alpha_neg)...
                * weighting('neg',delta_pos,delta_neg,pr(i));
        else
            objective = objective + utility(x(i), lambda, alpha_pos, alpha_neg)...
                * (weighting('neg',delta_pos,delta_neg,sum(pr(1:i)))- ...
                weighting('neg',delta_pos,delta_neg,sum(pr(1:i-1))));
        end
    end
end
end
```

3 Transformation

3.1 From Naïve to Sophisticated Strategy

The codes in this subsection generate the naïve strategy *"p_naive"* and the updated strategy in each round *"p_update"* when training the naïve one to a sophisticated strategy. Also, the CPT preference value for strategy *"p_naive"* and *"p_update"* is given by *"v"*.

To show how to train the naïve strategy into a sophisticated one, we derive the naïve strategy *"p_naive"* first: at each node *(t0, x0)*, we seek a sequence of actions that optimize the function *"objective"* at node *(t0, x0)* by using the MATLAB function *"fmincon"*, and only implement the action at node *(t0, x0)*.

```
% naive strategy
p_naive = zeros(1,(T+1)*(T+2)/2-(T+1));
for i = 1:T
    % initial time t0
    t0 = i-1;
    % number of nodes in the tree
    nodes_num = (T-t0+1)*(T-t0+2)/2;
    % number of strategies in the tree
    p_num = nodes_num-(T-t0+1);
    for n = 1:i
        % initial state
        x0 = (i-1)-2*(n-1);
        % solving for optimal strategy at (t0, x0)
        problem = createOptimProblem('fmincon','x0',ones(1,p_num),'objective',@(p) -
objective(lambda, alpha_pos, alpha_neg, delta_pos, delta_neg, t0, x0, T,
p),'lb',zeros(1,p_num),'ub',ones(1,p_num),'options',optimset('Algorithm','sqp'));
        p = run(gs,problem);
        p_naive((i-1)*i/2+n) = p(1);
    end
end
```

Then at each round *iter*, we revisit the optimization problem *"objective"* at each node *(t0, x0)* given all other nodes adopting stopping strategies derived at the last round (*iter-1)*, that is, *"p_update(iter-1, :)"*, and update the stopping strategy at node *(t0, x0)* in *"p_update(iter, :)"*. The corresponding CPT preference value *"v(iter)"* for each updated strategy *"p_update(iter, :)"* is calculated by directly inputting the strategy *"p_update(iter, :)"* in function *"objective"*. Figure 2 plots the naïve strategy *"p_naive"* and its updated version *"p_update"* at each round under $T = 5$, *alpha_pos* = *alpha_neg* = 0.9, *delta_pos* = *delta_neg* = 0.5, *lambda* = 1.5; and Figure 3 under $T = 5$, *alpha_pos* = *alpha_neg* = 0.5, *delta_pos* = *delta_neg* = 0.9, *lambda* = 1.5.

```
% revisit the problem for each node given other strategy fixed
p_update = zeros(T,(T+1)*(T+2)/2-(T+1));
v = zeros(1,T);
p_update(1,:) = p_naive;
v(1) = objective(lambda, alpha_pos, alpha_neg, delta_pos, delta_neg, 0, 0, T, p_update(1,:));
for iter = 2:T    % at most T-1 iterations
    p_update_iter = zeros(1,(T+1)*(T+2)/2-(T+1));
    update_nodes_num = (T-iter+1)*(T-iter+2)/2;
    p_update_iter(update_nodes_num+1:end) = p_update(iter-1,update_nodes_num+1:end);
```

```
    for i = 1:T-iter+1
        t0 = i-1;     % initial time t0
        for n = 1:i
            x0 = (i-1)-2*(n-1); % initial state
            node_index = t0*(t0+1)/2 + n;
            % last updated strategy from time t0+1 to T
            p_fixed = [];
            for layer = 1:T-1-t0
                p_fixed= [p_fixed, p_update(iter-1,node_index+i:node_index+i+layer)];
                node_index = node_index+i+layer;
            end
            % solving optimal strategy at (t0,x0) given following strategies fixed
            problem = createOptimProblem('fmincon','x0',0.5,'objective',@(p0) -
objective(lambda, alpha_pos, alpha_neg, delta_pos, delta_neg, t0, x0, T, [p0,
p_fixed]),'lb',0,'ub',1,'options',optimset('Algorithm','sqp'));
            p0 = run(gs,problem);
            p_update_iter((i-1)*i/2+n) = p0;
        end
    end
    p_update(iter,:) = p_update_iter;
    v(iter) = objective(lambda, alpha_pos, alpha_neg, delta_pos, delta_neg, 0, 0, T,
p_update(iter,:));
end
```

3.2 Without Randomization

In this subsection, we extend the previous examples by considering the strategies without randomization. This means that the agent can only choose a probability of 1 or 0 to be her action at each node. Likewise, we still derive the naïve strategy *"p_naive"* first and update the strategy round by round through reoptimizing the strategy at each node given others remain fixed. The only difference is that the MATLAB function *"surrogateopt"*, instead of *"fmincon"*, is used to solve the integer-valued optimization problem. Figure 4 and 5 show the training process from the naïve strategy without randomization to the sophisticated strategy without randomization under T = 5, *alpha_pos* = *alpha_neg* = 0.9, *delta_pos* = *delta_neg* = 0.5, *lambda* = 1.5 and T = 5, *alpha_pos* = *alpha_neg* = 0.5, *delta_pos* = *delta_neg* = 0.9, *lambda* = 1.5 respectively.

```
% naive strategy
p_naive = zeros(1,(T+1)*(T+2)/2-(T+1));
for i = 1:T
    t0 = i-1;
    nodes_num = (T-t0+1)*(T-t0+2)/2;    % number of nodes in the tree
    p_num = nodes_num-(T-t0+1);         % number of strategies in the tree
    for n = 1:i
        x0 = (i-1)-2*(n-1);
        rng default
        intcon = 1:p_num;
        p = surrogateopt(@(p) -objective(lambda, alpha_pos, alpha_neg, delta_pos,
delta_neg, t0, x0, T, p),zeros(1,p_num),ones(1,p_num),intcon);
        p_naive((i-1)*i/2+n) = p(1);
    end
end

% revisit the problem for each node given other strategy fixed
p_update = zeros(T,(T+1)*(T+2)/2-(T+1));
v = zeros(1,T);
p_update(1,:) = p_naive;
v(1) = objective(lambda, alpha_pos, alpha_neg, delta_pos, delta_neg, 0, 0, T, p_update(1,:));
for iter = 2:T    % at most T-1 iterations
    p_update_iter = zeros(1,(T+1)*(T+2)/2-(T+1));
    update_nodes_num = (T-iter+1)*(T-iter+2)/2;    % only update strategy from time 0 to T-
iter
    p_update_iter(update_nodes_num+1:end) = p_update(iter-1,update_nodes_num+1:end);
    for i = 1:T-iter+1
        t0 = i-1;     % initial time t0
        for n = 1:i
            x0 = (i-1)-2*(n-1); % initial state
            node_index = t0*(t0+1)/2 + n;
            % last updated strategy from time t0+1 to T
            p_fixed = [];
            for layer = 1:T-1-t0
                p_fixed= [p_fixed, p_update(iter-1,node_index+i:node_index+i+layer)];
                node_index = node_index+i+layer;
            end
            % solving optimal strategy at (t0,x0) given following strategies fixed
            rng default
            intcon = 1;
            p0 = surrogateopt(@(p0) -objective(lambda, alpha_pos, alpha_neg, delta_pos,
delta_neg, t0, x0, T, [p0, p_fixed]),0,1,intcon);
```

```
                p_update_iter((i-1)*i/2+n) = p0;
            end
        end
        p_update(iter,:) = p_update_iter;
        v(iter) = objective(lambda, alpha_pos, alpha_neg, delta_pos, delta_neg, 0, 0, T,
p_update(iter,:));
end
```

3.3 Start with Arbitrary Strategy

The other extension is that we start with an arbitrary strategy, not the naïve one, and update it based on strategic reasoning. Here we suppose the agent starts with an initial strategy *"p_initial"*, not *"p_naive"*, where the stopping probability at all nodes are equally 0.5, then using the same codes as in the naïve one, *"fmincon"* is used to optimize the function *"objective"* and update the strategy *"p_update"*. Figure 6 and 7 show how an arbitrary strategy *"p_initial"* is trained into the sophisticated strategy round by round under $T = 5$, *alpha_pos* = *alpha_neg* = 0.9, *delta_pos* = *delta_neg* = 0.5, *lambda* = 1.5 and $T = 5$, *alpha_pos* = *alpha_neg* = 0.5, *delta_pos* = *delta_neg* = 0.9, *lambda* = 1.5 respectively.

```
% initialization
p_initial = 0.5*ones(1,(T+1)*(T+2)/2-(T+1));
p_update = zeros(T+1,(T+1)*(T+2)/2-(T+1));
p_update(1,:) = p_initial;
v = zeros(1,T);
v(1) = objective(lambda, alpha_pos, alpha_neg, delta_pos, delta_neg, 0, 0, T, p_update(1,:));
% iteration
for iter = 1:T    % at most T iterations
    p_update_iter = zeros(1,(T+1)*(T+2)/2-(T+1));
    update_nodes_num = (T-iter+1)*(T-iter+2)/2;
    if iter ~= 1
        p_update_iter(update_nodes_num+1:end) =
p_update(iter,update_nodes_num+1:end);
    end
    for i = 1:T-iter+1
        t0 = i-1;     % initial time t0
        for n = 1:i
            x0 = (i-1)-2*(n-1); % initial state
            node_index = t0*(t0+1)/2 + n;
            % last updated strategy from time t0+1 to T
            p_fixed = [];
```

```
            for layer = 1:T-1-t0
                p_fixed= [p_fixed, p_update(iter,node_index+i:node_index+i+layer)];
                node_index = node_index+i+layer;
            end
            % solving optimal strategy at (t0,x0) given following strategies fixed
            problem = createOptimProblem('fmincon','x0',0.5,'objective',@(p0) -
objective(lambda, alpha_pos, alpha_neg, delta_pos, delta_neg, t0, x0, T, [p0,
p_fixed]),'lb',0,'ub',1,'options',optimset('Algorithm','sqp'));
            p0 = run(gs,problem);
            p_update_iter((i-1)*i/2+n) = p0;
        end
    end
    p_update(iter+1,:) = p_update_iter;
    v(iter+1) = objective(lambda, alpha_pos, alpha_neg, delta_pos, delta_neg, 0, 0, T,
p_update(iter+1,:));
end
```
